# Perceptions and Satisfaction With the Use of Digital Medical Services in Urban Older Adults of China: Mixed Methods Study

**DOI:** 10.2196/48654

**Published:** 2024-09-20

**Authors:** Ning Wang, Siyu Zhou, Zhuo Liu, Ying Han

**Affiliations:** 1 School of Management Shanxi Medical University Jinzhong China; 2 School of Public Health Hangzhou Normal University Hangzhou China

**Keywords:** digital medical services, older adults, Technology Acceptance Model, perception, behavioral intention, satisfaction

## Abstract

**Background:**

In an aging and information-driven society, older adults have distinct perceptions of and specific demands for digital medical services. It is essential for society to understand these needs and develop a more thoughtful approach to digital health care.

**Objective:**

This study aims to evaluate the behavioral intention and satisfaction of older adults with digital medical services by identifying the perceived factors and the pathways through which these factors influence their behavior.

**Methods:**

This study used a mixed methods approach, combining qualitative and quantitative analyses. A focus group interview was conducted with 30 randomly selected older adults, and the interviews were transcribed verbatim and coded using grounded theory. In addition, 876 valid questionnaires were collected to describe older adults’ perceptions of and satisfaction with digital medical care. Then, t tests and ANOVA were used to explore differences among various demographic groups, while hierarchical multiple regression was conducted to identify the factors most closely related to satisfaction. Structural equation modeling was used to identify multiple mediating effects.

**Results:**

The qualitative study identified the core category of “medical service relief and transformation paths for older adults in the context of digital reform.” Quantitative analysis revealed that more than half of the older adults were satisfied with digital medical services, and behavioral intentions were higher among those with higher incomes and education levels. Structural equation modeling confirmed that external variables, such as digital skills training, positively influenced perceived ease of use (β=.594, *P*<.001), perceived usefulness (β=.544, *P*<.001), and promoted digital medical behavioral intentions (β=.256, *P*<.001), while also reducing perceived risk (β=–.295, *P*<.001). Additionally, perceived ease of use (β=.168, *P*<.001) and perceived usefulness (β=.508, *P*<.001) positively impacted behavioral intention, whereas perceived risk (β=–.05, *P*=.037) exerted a negative influence. Furthermore, behavioral intention (β=.641, *P*<.001) significantly and positively affected older adults’ satisfaction with digital medical care. The mediation test identified 4 significant paths: (1) external variables → perceived ease of use → behavioral intention (effect size of 13.9%); (2) external variables → perceived usefulness → behavioral intention (effect size of 38.4%); (3) external variables → perceived ease of use → perceived usefulness → behavioral intention (effect size of 10.1%); and (4) a direct effect (35.5%) from external variables to behavioral intention.

**Conclusions:**

Based on the study’s findings, addressing the needs of older adults and enhancing perceived usefulness are the most effective ways to encourage the use of digital health care devices. Community support plays a crucial role in helping older adults integrate into digital health care, and adapting the design of services and products to suit their needs improves their perceptions of digital health care. This, in turn, promotes usage behavior and satisfaction, while the negative impact of perceived risk remains minimal.

## Introduction

### Background

According to data from the seventh national census, China has 260 million people aged 60 or older, accounting for 18.7% of the country’s population, which is significantly higher than the global average of 12.8%. Aging has become a key concern for society, and the ongoing transformation of this issue continues to place significant pressure and challenges on China. With the rapid advancement of internet technology, “Internet Plus Service” has been applied across various sectors, creating a synergistic effect that benefits the public. When integrated with traditional health care models, the digital approach of “Internet Plus Healthcare,” combined with resident services, has achieved significant success in multiple areas of society. However, older adults have increasingly become “information islands,” marginalized from the digital society [1].

In the current era of rapidly advancing information and communications technology, older adults are increasingly digitally excluded [2]. In China, individuals over 60 years of age make up 41.9% of all noninternet users [3], and this group represents a marginal audience with a low willingness to use digital medical services [4]. Their reluctance is often driven by fear of the unfamiliar features of digital and mobile products. The combined influence of various factors, including personal, family, social, and technological aspects [5], has resulted in older adults struggling to efficiently access health information and services, contributing to the emergence of the “health digital divide” [6]. Compared with younger age groups, older adults must invest more effort and time to overcome the greater challenges they face when familiarizing themselves with internet technologies, such as digital medical services [7]. A study found that digital medical utilization in Jordan is significantly influenced by demographic factors, particularly age (due to physiological changes) and gender [8]. Previous research also suggests that healthy older adults are more likely to use the internet compared with those with medical conditions [7]. As body functions deteriorate with age, older adults often experience physical impairments such as hearing and vision loss [9,10], memory decline [11], reduced fine motor control [12], and cognitive decline [13], all of which affect their willingness to adopt digital medical services [14]. Concerns about the security of internet information further diminish older adults’ willingness to use it. They face numerous barriers when attempting to utilize digital medical services [15], leading to technology-related anxiety [16]. According to Heart and Kalderon [17], the factors influencing health information technology utilization among aging users can be summarized into 6 dimensions: usefulness, ease of use, technology-related issues, user characteristics, social factors, and convenience measures [17].

Currently, the digital medical health care and service system for older adults in China is not yet well developed. Older adults who lack digital literacy are unable to benefit from the convenience of digital services, which in turn reduces the efficiency of medical treatment for this group. Age-related declines in physical functions, such as vision, hearing, and mobility, contribute to weaker adaptability and lower sensitivity to digital health care. Additionally, older adults may experience behavioral disorientation and diminished perception of health services during medical visits. Declining health care satisfaction among older adults has inevitably led to digital health care inequities [18]. The growing injustice in digital health, known as the “health digital divide,” has significantly contributed to the underutilization of health care services by older adults in their daily care processes. To address this issue, the State Council of the People’s Republic of China has emphasized the importance of “accelerating the construction of a digital society.” This initiative aims to promote the use of digital services in medical, health care, and pension services, with the goal of continuously improving the public’s sense of access. A series of policies have been introduced, focusing on high-frequency health care issues for older adults in the digital environment, with an emphasis on enhancing daily medical care as a key aspect of improving people’s livelihoods.

This study focuses on Hangzhou City in Zhejiang Province, known as the “first city of digital governance in China.” As a rapidly maturing megacity, Hangzhou boasts a high level of internet development [19]. Numerous internet technology enterprises, spearheaded by Alibaba, have supported Hangzhou’s digital growth. Additionally, Hangzhou’s strong influence has contributed to a level of digital governance in the Yangtze River Delta region that is significantly higher than the national average [20]. Numerous studies on digital health care and digital infrastructure development have cited Hangzhou as a model for both national and global urban digital governance [21,22].

Hangzhou City, recognized as a national role model for older adult care services, is actively implementing the national strategy to address population aging by making smart products more intelligent, practical, accessible, and suitable for older adults. While Hangzhou has made significant strides in exploring medical innovations, the system for digitally accessing medical services for older adults still requires further development. The purpose of this study is to investigate the current perception and satisfaction with digital medical services among older adults in Hangzhou through a survey. It aims to explore the primary perceptual reasons why older adults struggle to adapt to digital medical services amid the existing “health digital divide” and to propose strategies to help them overcome these challenges. This research holds significant practical value for advancing digital health care transformation, providing insights for digital reform in other areas, and enhancing its impact on digital development in China and neighboring countries. Additionally, this study integrates perceived risk and satisfaction into the Technology Acceptance Model (TAM), examining the potential relationships between these variables, which could contribute theoretically to the further refinement of the model.

### Theories and Hypotheses

**Figure 1:**
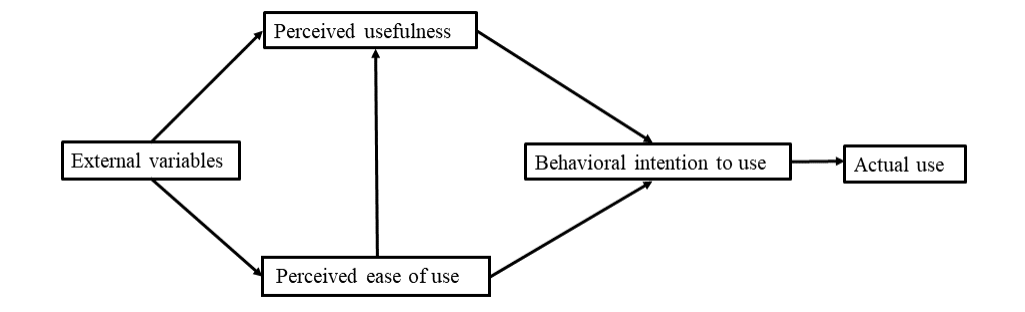
The Technology Acceptance Model revised by Davis in 1996.

The TAM, depicted in Figure 1, is the most established and robust theoretical model in the field of information systems. Developed from the Theory of Reasoned Action and first proposed by Davis in 1989 [23], the TAM has undergone revisions in 1993 and 1996, evolving into the most widely used and generalized model. The modified TAM includes 3 core concepts: perceived usefulness, perceived ease of use, and behavioral intention to use.

**Figure 2:**
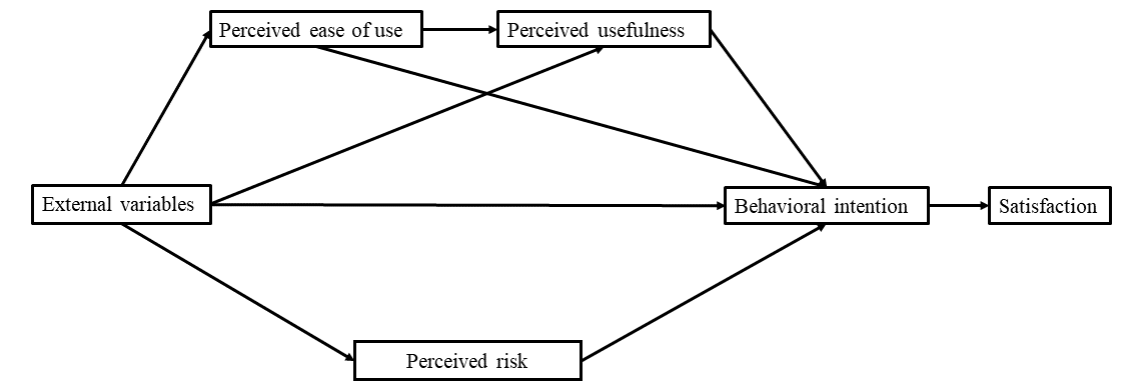
Satisfaction model of digital medical service utilization for older adults.

The TAM provides strong predictive and explanatory power regarding users’ acceptance behavior. This study focuses on behavioral intention and satisfaction factors influencing the use of digital medical services among the aging population, including new technologies such as online booking and e-payment. Given that this population often harbors varying degrees of distrust toward digital technology, it is essential to consider the perceived risks associated with new technologies when developing new models [24]. Therefore, building on the TAM, this paper integrates the perceived risk associated with digital medical services for older adults into the model, using their satisfaction with these services as the outcome variable. The study examines the interactive relationships between perceptual elements, analyzes how each element impacts satisfaction, and aims to elucidate the influence of perceived health care utilization among urban older adults from the demand perspective. The final model is presented in Figure 2.

External variables include system characteristics, interface features, individual differences, and other factors [25]. Systematic assistance from digital devices can facilitate easier access to useful information, and the more straightforward this access, the more convenient it is for the user [26]. Additionally, well-designed interface features of electronic devices can reduce the user’s burden, thereby influencing perceived ease of use, which in turn affects behavioral intentions. If older adults perceive digital devices as highly convenient for their lives, their desire to access digital health care is further enhanced [27]. Additionally, if they receive education about digital medical services and guidance from volunteers, family, and friends during the use of these services, their perceived usefulness will improve significantly, thereby increasing their behavioral intention to use digital medical services. This study considers external variables such as social environment factors, including training older adults in the use of digital equipment and the distribution of promotional materials. The use of online applications or self-service machines for registration and bill payment can reduce queuing time and provide additional conveniences. Individual differences are used as a control variable. According to Davis [23], external variables influence users’ perceived ease of use of new technologies, and both external variables and perceived ease of use together determine perceived usefulness.

In this study, perceived usefulness refers to older adults’ belief that using digital health services will enhance their access to health care or facilitate obtaining health-related information. Perceived ease of use refers to how simple older adults find it to operate digital medical services, such as accessing instructions for using digital devices. Perceived ease of use has a significant and positive impact on perceived usefulness [23,28]. Martin et al [29] conducted a study on the adoption of mobile health (mHealth) apps among patients in community health service centers and found that both perceived ease of use and perceived usefulness positively influenced the behavioral intention to adopt these technologies. Lee’s [30] study confirmed this mediating effect, demonstrating that perceived ease of use influences the attitude toward using digital services by affecting perceived usefulness.

Bauer [31] defined perceived risk as “a combination of uncertainty plus seriousness of outcome involved.” In this study, perceived risk refers to older adults’ concerns about safety when using new technologies for digital medical services, which directly affects their widespread adoption. Older adults may worry about risks such as personal information leakage and insecure payments when using these services [24,32]. Without adequate digital health care training and publicity from governments and organizations, mistrust of digital medical services among older adults may increase. This heightened perception of risk can diminish their motivation to engage in digital health care.

Behavioral intention refers to the willingness of older adults to use digital medical services, reflecting their subjective intent. Satisfaction, by contrast, pertains to the level of contentment with the services before, during, and after a digital medical visit. This study posits that external variables, such as social environment and convenience, influence the behavioral intention of older adults by shaping their perceptions of digital health care. These perceptions, in turn, affect their satisfaction with digital health care services. Based on this hypothesis, the following propositions are made, informed by theory and existing research.

- H1: External variables positively influence perceived ease of use and perceived usefulness, as well as behavioral intention, while negatively influencing perceived risk.
- H2: Perceived ease of use and perceived usefulness positively affect behavioral intention, while perceived risk negatively affects behavioral intention.
- H3: Perceived ease of use, perceived usefulness, and perceived risk have parallel multiple mediating effects between external variables and behavioral intention.
- H4: Behavioral intention is influenced through a chain mediation process, where perceived ease of use affects perceived usefulness, which in turn influences perceived risk, ultimately impacting behavioral intention.
- H5: Behavioral intention positively influences satisfaction with digital health care among older adults.

## Methods

### Participants

In this study, a multistage sampling method was applied. First, 4 administrative districts were randomly selected from the 8 main urban areas of Hangzhou. Next, a community health service center was randomly chosen from each of these districts. Finally, older adults aged 60 and over were numbered using the eHealth medical record database of each selected community health service center. As many as 5-8 individuals were randomly selected by a computer program, and these older adults were contacted by a team of family physicians. In each community, at least five participants aged 60 years or older, who had experience with digital health care, were included in focus group interviews. Additionally, for the questionnaire survey, the computer program randomly selected 250 older adults with self-directed health care behaviors from each community health service center. Before the official survey, a presurvey was conducted with 50 older adults. Based on their feedback, the questionnaire was refined to ensure clarity and ease of understanding, with adjustments made to the questions and page layout to better suit older adults (Multimedia Appendix 1).

All survey participants were trained in advance to familiarize themselves with the interview outline and questionnaire content, ensuring a thorough understanding of their meaning. They explained the purpose and significance of the study to the participants and conducted the questioning and survey only after obtaining informed consent. Ideally, the questionnaires were completed by the older adults themselves. For those who had difficulty filling out the questionnaires, the surveyors read each question aloud and recorded the participants’ responses to ensure that the questionnaires were completed accurately and thoroughly.

### Semistructured Interviews

In this study, the interview outline was initially developed based on literature research and then revised through expert consultation to finalize the outline. Grounded theory was used as the methodological framework, and NVivo 11.0 (Lumivero) was used for data analysis, including open coding, axial coding, and selective coding of the raw data. Memos and interview outlines were also prepared as part of the analysis process (Textbox 1).

**Interview outlines.**

- Basic digital health perception
  - What are your health needs? Is it possible to solve this through digital methods?

- Digital medical use experience
  - Which functions of digital devices have you used, and can you tell us about your experience with and feelings about using them?
  - What are the advantages and disadvantages of digital medical services compared with manual services?

- Digital medical use dilemma
  - Do you have any difficulties using it?
  - Do you have any concerns or worries about using digital equipment for registration, and billing, among others?
  - Have you received help from family, friends, the community, volunteers, or medical personnel?

- Recommendations for digital medical services
  - What can be improved in digital medical services?

**Figure 3:**
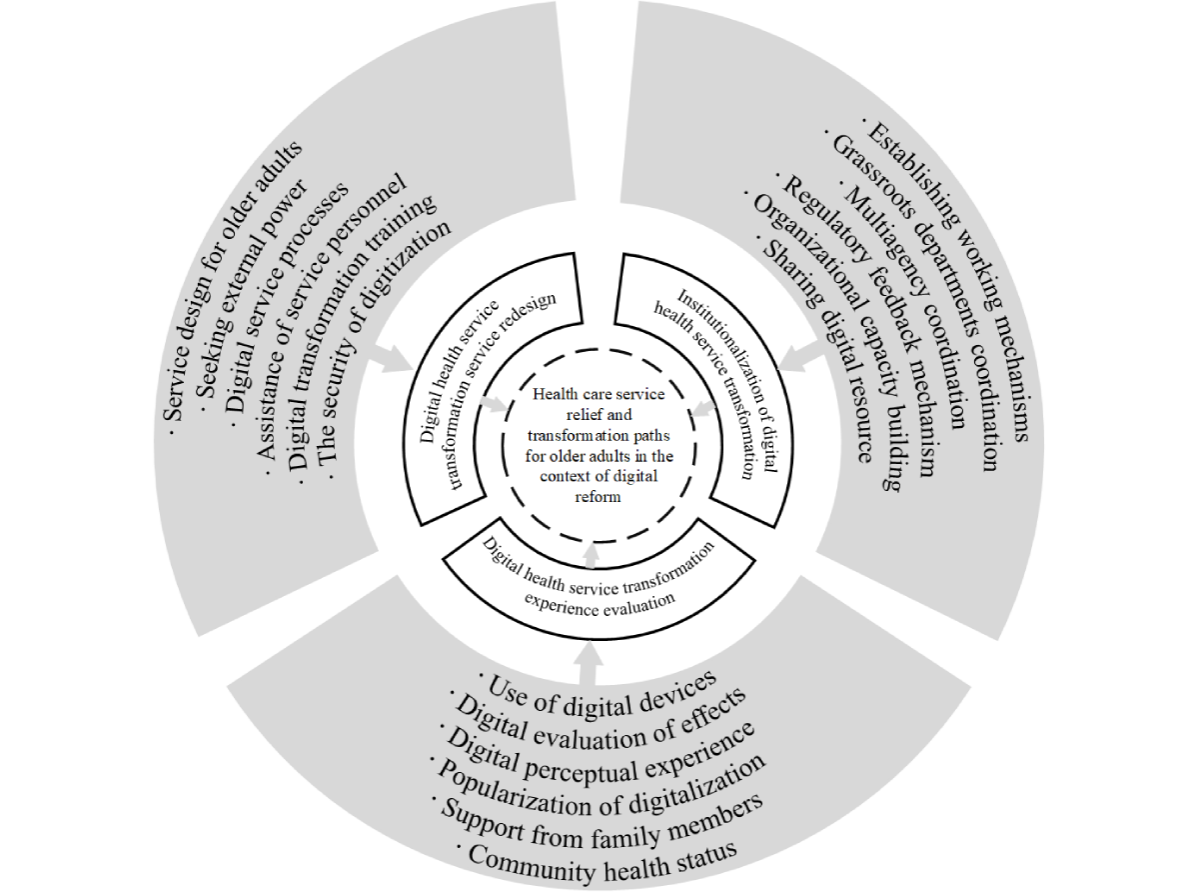
The results of 3-level coding.

We conducted semistructured interviews with 30 older adults aged 60 years and over. This sample size met the requirements of grounded theory research, and data saturation was deemed achieved when the interviewer began hearing repeated narratives. The interview recordings and textual materials were compiled into 110,000 words of raw data. Two-thirds of these data were randomly selected for analysis, while the remaining one-third were used to test theoretical saturation. Following grounded theory [33], the study conducted open coding and conceptualization of the original text, analyzing and categorizing each word. This process resulted in 668 conceptual labels from the open coding of the data. After eliminating invalid and repetitive concepts, 125 valid concepts and 18 categories were identified. To further clarify the relationships between these categories, axial coding was used to group and recategorize the main and subcategories based on their logical relationships. The core categories were summarized from the main categories, and storylines were developed to capture the key findings within a broader theoretical context, verifying these relationships with all available information. After repeatedly comparing and analyzing the connections among the major categories, the core category was refined to present a comprehensive view of the case, titled “Medical Service Relief and Transformation for Older Adults in the Context of Digital Reform.” This analysis focused on the barriers to accessing and using digital health care from a perceptual perspective, aiming to bridge the “health digital divide” and promote health equity. Figure 3 illustrates the storyline, main categories, and subcategories. Theoretical saturation occurs when no new concepts or categories emerge during the data analysis process [34]. By continuing to code the remaining one-third of the interview texts, we found that no new concepts or relationships were generated. This indicates that the coding process for this study is nearly complete, and theoretical saturation has been achieved, providing a certain degree of realistic explanatory power. The qualitative research effectively examines the influencing factors, satisfaction, suggestions, expectations, and future prospects for digital medical service utilization among older adults in Hangzhou, offering valuable insights for questionnaire design.

### Quantitative Analysis

Following the qualitative research, a questionnaire was developed and administered by trained investigators who adhered to uniform protocols. The purpose and significance of the study were explained to eligible senior citizens, and their consent was obtained before beginning the survey. To ensure accuracy, each respondent’s records were reviewed individually. We then analyzed the collected data, removed invalid questionnaires, and addressed dummy variables for categorical data. Reliability and validity tests were conducted to ensure the quality of the data. The Cronbach α coefficient for the overall scale in this study was 0.914, and the Cronbach α values for all dimensions were greater than 0.8, indicating that the reliability of the questionnaire was excellent.

First, the scale designed in this study demonstrates good content validity, based on references to numerous studies and modifications made to the questionnaire content following expert consultation. Second, the Bartlett test and the Kaiser-Meyer-Olkin test were conducted, with a Kaiser-Meyer-Olkin value of 0.933 (*P*<.001), indicating that the data are highly suitable for factor analysis. Third, confirmatory factor analysis was performed using Amos 26.0 (IBM Corp.). The overall fit indices of the model were acceptable, suggesting good structural validity (*χ*^2^/*df*=4.957, *df*=303, root-mean-square error of approximation 0.067, normed fit index 0.926>0.9, relative fit index 0.914>0.9, incremental fit index 0.940>0.9, Tucker-Lewis index 0.930>0.9, and comparative fit index 0.940>0.9). Fourth, the average variance extracted for each latent variable was greater than 0.5, and the composite reliability was greater than 0.8, indicating ideal convergent validity (Table 1; also see Textbox 1). Fifth, as shown in Table 2, most correlation coefficients were less than the square root of the average variance extracted, suggesting that there is a sufficient level of differentiation between the latent variables. This indicates that the discriminant validity of the scale’s data is acceptable. In summary, the questionnaire demonstrates good validity.

**Table 1:** Questionnaire items and results of reliability and validity tests.

Dimensions^a^	Cronbach α	Average variance extracted	Composite reliability	References
External variables	0.856	0.503	0.847	[35-37]
Perceived ease of use	0.889	0.787	0.917	[28,38-40]
Perceived usefulness	0.896	0.742	0.896	[28,38-40]
Perceived risk	0.841	0.549	0.827	[24,41,42]
Behavioral intention	0.943	0.884	0.942	[28,38,39]
Satisfaction	0.946	0.662	0.940	[40,43,44]

^a^See Textbox 2 for scale items.

**Scale items of the questionnaire.**

- External variables
  - T1: If digital devices improve friendliness (eg, longer operation time, simplified interaction interface and flow, and enlarged fonts), it will increase my willingness to use.
  - T2: The community offers training on the use of digital medical devices, and I would like to attend.
  - T3: I would like to read and study the digital medical information provided by the community.
  - T4: I think self-service registration (self-service machines or cell phones) is more convenient than manual registration.
  - T5: I think it is more convenient to make an appointment than to wait for a consultation on-site.
  - T6: I think self-service payment (self-service machines or cell phones) is more convenient than tollbooth.

- Perceived ease of use
  - T7: I can use my smartphone proficiently.
  - T8: I can make online appointments and payments.
  - T9: I can operate the self-service machines in the hospital.

- Perceived usefulness
  - T10: Digital access can save time.
  - T11: Digital access helps me get more information about medical care in advance.
  - T12: It is easier to adapt to society if you have digital medical skills.

- Perceived risk
  - T13: I am concerned that third-party platforms used in the digital medical process (eg, WeChat, various apps) may disclose personal information.
  - T14: I am concerned about the security of online and self-service machine payment.
  - T15: I am concerned that there is a haphazard fee for doctor’s office billing.
  - T16: I am concerned about the accuracy of the self-service report retrieval.

- Behavioral intention
  - T17: I am willing to accept digital access to medicine.
  - T18: I would like to learn about the use of digital medical equipment.
  - T19: I would like to use digital medical equipment.

- Satisfaction
  - T20: Various ways of digital appointment registration.
  - T21: Guidance on the use of digital medical equipment.
  - T22: Online doctor appointment.
  - T23: Call the information indicator screen in the waiting room.
  - T24: Pay for medical treatment in the doctor’s office without paying at the tollbooth.
  - T25: Self-service payment (mobile phone/self-service machine).
  - T26: Self-service report retrieval (mobile phone/self-service machine).
  - T27: The whole process of digital medical treatment.

**Table 2:** Discriminant validity^a^.

Dimensions	External variables	Perceived ease of use	Perceived risk	Perceived usefulness	Behavioral intention	Satisfaction
External variables	*0.503*	—^b^	—	—	—	—
Perceived ease of use	0.594	*0.787*	—	—	—	—
Perceived risk	–0.295	–0.175	*0.549*	—	—	—
Perceived usefulness	0.688	0.565	–0.203	*0.742*	—	—
Behavioral intention	0.721	0.617	–0.258	0.79	*0.844*	—
Satisfaction	0.462	0.395	–0.165	0.506	0.641	*0.662*
Square root of average variance extracted	0.709	0.887	0.741	0.861	0.919	0.814

^a^The diagonal cell value (italicized) is the average variance extracted.

^b^Not available.

The questionnaire uses a 5-point Likert scale, where 1 denotes “strongly disagree,” 2 denotes “disagree,” 3 denotes “neutral,” 4 denotes “agree,” and 5 denotes “strongly agree.” This scale is used to assess older adults’ feedback on digital medical services, as shown in Table 2.

The core formula for scoring for each dimension is as follows:

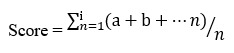



The questionnaire includes the following: (1) basic demographic characteristics, such as gender, age, education level, marital status, residency, former occupation, and frequency of medical treatment, to provide descriptive statistical analysis of the sample for the empirical study; (2) a survey scale for digital health care service utilization among urban older adults, covering external variables, perceived ease of use, perceived usefulness, perceived risk, and behavioral intentions; and (3) satisfaction evaluation of digital medical services, which includes ratings for each stage of digital access to care.

We identified the primary concerns of older adults and synthesized findings from previous studies and qualitative research to design questionnaire items corresponding to each dimension. The “external variables” were designed to assess the impact of external factors on the willingness to use and satisfaction with digital medical devices among older adults. These factors include age-adapted equipment (T1), community guidance on use (T2 and T3), and convenience conditions (T4, T5, and T6). The “perceived ease of use” dimension examines difficulties encountered with devices (smartphones T8 and kiosks T9) and processes (registration and payment T4) during medical care, which may affect willingness to use and satisfaction. The “perceived usefulness” dimension evaluates the role and value of digital health care services for older adults from 3 perspectives: time saving (T10), knowledge acquisition (T11), and social adaptation (T12). This helps in identifying areas for service improvement and upgrading. The “perceived riskiness” dimension investigates the risks that concern older adults, including privacy protection (T13), payment security (T14), cost settlement (T15), and report accuracy (T16). Addressing these risks in software design can enhance trust in digital health care. Finally, the “behavioral intentions” dimension analyzes acceptance (T15), learning (T16), and usage (T17) processes to identify barriers to digital health care service utilization by older adults. This provides insights for future intervention development to improve engagement and satisfaction.

According to the sample estimation method proposed by Kendall [45], which suggests having 10-20 samples per questionnaire item, and accounting for the questionnaire recovery rate and efficiency, the sample size should be increased by 20%. Using the formula 27 questions items × 20 × (1 + 20%) = 648, the study requires a minimum of 648 samples. As a result of the complex analysis of the data using multivariate statistical methods, the sample size was increased to 1000 to ensure more robust statistical parameters. Data entry was performed by 2 individuals using 2 separate machines. Out of the 1000 questionnaires distributed, 876 valid questionnaires were recovered, resulting in an effective recovery rate of 87.60%.

In this study, descriptive analyses were performed to understand the basic demographic information and perceptions of digital access among the older adults interviewed. Additionally, difference analyses were conducted to determine whether there were variations in core explanatory variables across different genders, ages, educational levels, and other demographic characteristics. Hierarchical regression analysis was used to determine whether including core explanatory variables—such as external variables, perceived ease of use, perceived usefulness, perceived risk, and behavioral intention—was meaningful for refining the model. Finally, structural equation modeling was used to test the multiple mediating effects of perceived usefulness, perceived ease of use, and perceived risk on the relationship between external variables and behavioral intention, as well as the chain mediating effects of perceived ease of use and perceived usefulness.

### Ethics Approval

This study was approved by the Hangzhou Normal University Ethics Committee (research ethics committee number 2021-1147). Participants were informed about the use of their data before both the interview and questionnaire began and were given the right to be informed and to withdraw at any time. All data were anonymized to protect participant privacy. As the survey posed no potential harm to respondents, we provided facial tissues, towels, and other household items as tokens of appreciation for their participation and cooperation.

## Results

### Participant Characteristics

Descriptive analyses were conducted to assess the demographic characteristics, perceptions of digital medical care, and current satisfaction levels among the surveyed older adults. Table 3 presents the demographic information of the respondents. The majority had an education level of junior high school (366/876, 41.8%). Additionally, over one-half (478/876, 54.6%) of older adults reported that declining physical function impacted their use of digital devices. Their preferred registration methods were, in order, manual window registration, self-service machine registration, and online registration. Feedback from older adults indicated several issues with digital medical equipment, including overly complex interfaces and processes, short time limits, small font sizes, inconsistent system updates across hospitals, and frequent equipment failures. These issues increase the difficulty of using digital health care devices and may serve as significant barriers to access and utilization for older adults, exacerbating gaps in access to health information and widening the “health digital divide.”

**Table 3:** Participant demographic data (N=876).

Characteristics			Participants n (%)
**Gender**			
	Male	358 (40.9)	
	Female	518 (59.1)	
**Age (years)**			
	60-69	433 (49.4)	
	70-79	282 (32.2)	
	80-89	151 (17.2)	
	≥90	10 (1.1)	
**Education**			
	Primary and below	125 (14.3)	
	Junior high school	366 (41.8)	
	Technical secondary school/senior high school	243 (27.7)	
	Junior college/bachelor and above	142 (16.2)	
**Marriage**			
	Married	724 (82.6)	
	Unmarried	19 (2.2)	
	Bereaved spouse	133 (15.2)	
**Living conditions**			
	Living with spouse	400 (45.7)	
	Living with spouse and children	288 (32.9)	
	Living alone	141 (16.1)	
	Others	47 (5.4)	
**Monthly disposable income (CNY^a^)**			
	<1000	19 (2.2)	
	1001-3000	124 (14.2)	
	3001-5000	483 (55.1)	
	5001-7000	179 (20.4)	
	>7000	71 (8.1)	
**Previous occupations**			
	Government offices	64 (7.3)	
	Enterprises and businesses	575 (65.6)	
	Private enterprise	127 (14.5)	
	Individuals	62 (7.1)	
	Others	48 (5.5)	
**Frequency of medical treatment in the past 6 months**			
	0	166 (18.9)	
	1-2	306 (34.9)	
	3-4	135 (15.4)	
	>5	269 (30.7)	
**Health insurance**			
	Urban employee medical insurance	431 (49.2)	
	Urban residents’ medical insurance	357 (40.8)	
	New rural cooperative medical care	35 (4.0)	
	Commercial health insurance	10 (1.1)	
	None	41 (4.7)	
	Others	2 (0.2)	
**Deterioration of physical function (eg, vision loss, hearing loss) that affects your ability to use digital medical devices**			
	Totally disagree	69 (7.9)	
	Comparatively disagree	95 (10.8)	
	Generally	234 (26.7)	
	Comparatively agree	395 (45.1)	
	Totally agree	83 (9.5)	
**Preferred appointment method**			
	Online	157 (17.9)	
	Self-service machines	323 (36.9)	
	Manual window	334 (38.1)	
	Doctor makes the next clinic appointment	19 (2.2)	
	Make a call	28 (3.2)	
	Others	15 (1.7)	
**The problems of digital medical equipment**			
	Time limit is too short	106 (12.1)	
	Too complicated interface and process	469 (53.5)	
	Font size too small	92 (10.5)	
	Others	209 (23.9)	

^a^1 CNY=US $0.14.

### Participants’ Perceptions and Behavioral Intentions of Digital Medical Care

Table 4 presents the descriptive analysis of scores for each influencing factor related to digital medical service utilization among urban older adults. The SD for all variables was above 0.5, indicating variability in the responses among the survey participants. After compiling the statistical data, the scores for the dimensions influencing the perception of digital medical service utilization among older adults, ranked from the highest to the lowest, were as follows: perceived usefulness (mean 3.78, SD 0.86), external variables (mean 3.68, SD 0.74), behavioral intention (mean 3.61, SD 0.97), perceived ease of use (mean 2.94, SD 1.01), and perceived risk (mean 2.80, SD 0.87). This indicates that while most older adults perceive digital health care access as beneficial, challenges such as operational difficulty and perceived risks hinder their effective use of digital access devices.

**Table 4:** Descriptive statistical analysis of influencing factors^a^.

Influencing factors	Range	Mean (SD)
External variables	1.00-5.00	3.68 (0.74)
Perceived usefulness	1.00-5.00	3.78 (0.86)
Perceived ease of use	1.00-5.00	2.94 (1.01)
Perceived risk	1.00-5.00	2.80 (0.87)
Behavioral intention	1.00-5.00	3.61 (0.97)

^a^Number of valid cases: 876.

### Participant Satisfaction With Each Aspect of Digital Medical Care

Table 5 shows the satisfaction evaluation of digital medical services and equipment at various stages of the diagnosis process. More than 60% of respondents expressed satisfaction with aspects such as registration methods (570/876, 65.1%), digital medical guidance (550/876, 62.8%), online doctor appointments (527/876, 60.2%), call information indicator screens in waiting rooms (584/876, 66.7%), payment at the doctor’s office (545/876, 62.2%), self-service payment (545/876, 62.2%), and self-service report retrieval (558/876, 63.7%).

**Table 5:** The constituent ratio of digital medical satisfaction (N=876).

Variables	Highly dissatisfied, n (%)	Dissatisfied, n (%)	Common, n (%)	Satisfied, n (%)	Highly satisfied, n (%)
Various ways of digital appointment registration	10 (1.1)	47 (5.4)	249 (28.4)	443 (50.6)	127 (14.5)
Guidance on the use of digital medical equipment	8 (0.9)	62 (7.1)	256 (29.2)	412 (47.0)	138 (15.8)
Online doctor appointment	11 (1.3)	51 (5.8)	287 (32.8)	396 (45.2)	131 (15.0)
Call information indicator screen in the waiting room	7 (0.8)	38 (4.3)	247 (28.2)	444 (50.7)	140 (16.0)
Pay for medical treatment in the doctor’s office without paying at the tollbooth	9 (1.0)	44 (5.0)	278 (31.7)	401 (45.8)	144 (16.4)
Self-service payment (mobile phone/self-service machine)	12 (1.4)	61 (7.0)	258 (29.5)	415 (47.4)	130 (14.8)
Self-service report retrieval (mobile phone/self-service machine)	13 (1.5)	63 (7.2)	242 (27.6)	429 (49.0)	129 (14.7)
The whole process of digital medical treatment	9 (1.0)	45 (5.1)	238 (27.2)	464 (53.0)	120 (13.7)

### Differences in Perceptions, Behavioral Intentions, and Satisfaction Between Groups

The *t* test (2-tailed) and ANOVA were used to explore differences in external variables, perceived ease of use, perceived usefulness, perceived risk, behavioral intention, and satisfaction among individuals with different characteristics (as shown in Table 6). Gender differences were not statistically significant in external variables (*P*=.97), perceived ease of use (*P*=.42), perceived usefulness (*P*=.11), perceived risk (*P*=.98), behavioral intention (*P*=.27), and satisfaction (*P*=.83). Older age and lower levels of education were associated with lower scores on external variables, perceived ease of use, perceived usefulness, behavioral intention, and satisfaction. However, the differences in age (*P*=.10) and education (*P*=.14) in perceived risk were not statistically significant. Married older adults had significantly higher scores on external variables (*P*<.001) and overall satisfaction (*P*=.03). Those living with their children scored higher in perceived ease of use and perceived risk of digital medical devices. This may be because living with children provides easier access to guidance, which reduces the difficulty of using digital devices, while reminders from children about fraud may heighten their awareness of potential risks. Conversely, older adults living alone with their spouses scored higher in the remaining dimensions. Older adults with higher incomes reported lower perceived risk and higher scores across other dimensions. This trend may be linked to higher education levels associated with greater income. Additionally, older adults with previous work experience in government positions scored higher in perceived usefulness and behavioral intention, potentially due to increased confidence in trying new technologies gained from their professional backgrounds. Those with frequent access to health care had higher scores on external variables, perceived usefulness, behavioral intention, and satisfaction, and lower scores on perceived risk. This likely results from their greater familiarity with digital access devices, leading to increased proficiency and a better appreciation of the convenience digital health care offers.

**Table 6:** Difference analysis of characteristics of older adults.

Variables		External variables	Perceived ease of use	Perceived usefulness	Perceived risk	Behavioral intention	Satisfaction	
**Gender**								
	Male, mean (SD)	3.68 (0.69)	3.00 (1.08)	3.72 (0.89)	2.80 (0.90)	3.57 (0.98)	3.71 (1.08)	
	Female, mean (SD)	3.69 (0.77)	2.97 (1.08)	3.82 (0.83)	2.80 (0.85)	3.64 (0.96)	3.70 (0.71)	
	*t* test^a^ (*df*)	–0.031 (874)	–0.802 (874)	–1.597 (874)	0.018 (874)	–1.112 (874)	0.216 (874)	
	*P* value	.97	.42	.11	.98	.27	.83	
**Age (years)**								
	60-69, mean (SD)	3.81 (0.76)	3.35 (1.04)	3.90 (0.44)	2.75 (0.86)	3.79 (0.97)	3.82 (0.74)	
	70-79, mean (SD)	3.64 (0.63)	2.68 (0.95)	0.72 (0.43)	2.78 (0.88)	3.58 (0.84)	3.67 (0.65)	
	80-89, mean (SD)	3.47 (0.75)	2.33 (0.95)	0.88 (0.72)	2.94 (0.90)	3.22 (1.07)	3.49 (0.67)	
	>90, mean (SD)	2.63 (0.94)	2.27 (1.15)	0.80 (0.25)	3.05 (0.82)	2.53 (0.65)	3.41 (0.78)	
	*F* test (*df*)	12.611 (872)	51.127 (872)	7.809 (872)	2.087 (872)	20.613 (872)	9.298 (872)	
	*P* value	<.001	<.001	<.001	.10	<.001	<.001	
**Education**								
	Primary and below, mean (SD)	3.41 (0.87)	2.37 (1.07)	3.64 (0.82)	2.92 (0.89)	3.20 (1.15)	3.55 (0.78)	
	Junior high school, mean (SD)	3.66 (0.69)	2.85 (0.99)	3.72 (0.84)	2.83 (0.85)	3.55 (0.90)	3.68 (0.70)	
	Technical secondary school/senior high school, mean (SD)	3.78 (0.71)	3.19 (0.99)	3.86 (0.86)	2.75 (0.85)	3.75 (0.93)	3.78 (0.65)	
	Junior college/bachelor and above, mean (SD)	3.81 (0.74)	3.28 (1.20)	3.93 (0.89)	2.70 (0.94)	3.91 (0.91)	3.78 (0.74)	
	*F* test (*df*)	7.348 (872)	21.357 (872)	4.156 (872)	1.845 (872)	12.516 (872)	3.787 (872)	
	*P* value	<.001	<.001	.006	.14	<.001	.01	
**Marriage**								
	Married, mean (SD)	3.73 (0.71)	3.02 (1.06)	3.81 (0.84)	2.80 (0.87)	3.67 (0.92)	3.74 (0.70)	
	Unmarried, mean (SD)	3.54 (0.91)	3.04 (1.28)	3.65 (1.13)	2.74 (1.04)	3.68 (1.12)	3.49 (1.15)	
	Bereaved spouse, mean (SD)	3.44 (0.81)	2.53 (1.07)	3.64 (0.86)	2.80 (0.88)	3.28 (1.14)	3.57 (0.69)	
	*F* test (*df*)	9.647 (873)	11.827 (873)	2.270 (873)	0.049 (873)	7.007 (873)	3.837 (873)	
	*P* value	<.001	<.001	.10	.95	.002	.03	
**Living conditions**								
	Living only with spouse, mean (SD)	3.81 (0.66)	2.98 (1.09)	3.90 (0.83)	2.72 (0.87)	3.77 (0.90)	3.78 (0.68)	
	Living with spouse and children, mean (SD)	3.65 (0.70)	3.05 (0.99)	3.67 (0.82)	2.90 (0.84)	3.56 (0.90)	3.74 (0.66)	
	Living alone, mean (SD)	3.52 (0.90)	2.66 (1.16)	3.75 (0.91)	2.74 (0.96)	3.45 (1.12)	3.59 (0.84)	
	Others, mean (SD)	3.32 (0.82)	2.82 (1.13)	3.48 (0.99)	3.05 (0.70)	3.09 (1.20)	3.27 (0.74)	
	*F* test (*df*)	9.383 (872)	4.584 (872)	6.526 (872)	4.475 (872)	7.895 (872)	7.716 (872)	
	*P* value	<.001	.003	<.001	.005	<.001	<.001	
**Monthly disposable income (CNY^b^)**								
	<1000, mean (SD)	3.07 (1.28)	2.56 (1.14)	3.28 (1.21)	3.01 (1.12)	2.72 (1.10)	3.02 (1.15)	
	1001-3000, mean (SD)	3.54 (0.73)	2.74 (1.06)	3.56 (0.80)	2.98 (0.86)	3.37 (0.94)	3.62 (0.77)	
	3001-5000, mean (SD)	3.69 (0.69)	2.87 (1.03)	3.76 (0.82)	2.81 (0.83)	3.63 (0.92)	3.71 (0.63)	
	5001-7000, mean (SD)	3.75 (0.74)	3.20 (1.04)	3.97 (0.82)	2.70 (0.92)	3.74 (0.98)	3.73 (0.74)	
	>7000, mean (SD)	3.92 (0.74)	3.26 (1.32)	3.97 (1.02)	2.62 (0.95)	3.84 (1.09)	3.96 (0.75)	
	*F* test (*df*)	4.389 (871)	5.984 (871)	6.316 (871)	2.737 (871)	8.000 (871)	4.078 (871)	
	*P* value	.003	<.001	<.001	.03	<.001	.004	
**Previous occupations**								
	Government offices, mean (SD)	3.75 (0.77)	3.01 (1.11)	3.88 (0.85)	2.62 (0.90)	3.88 (0.78)	3.65 (0.74)	
	Enterprises and businesses, mean (SD)	3.73 (0.71)	2.98 (1.09)	3.86 (0.82)	2.77 (0.86)	3.68 (0.96)	3.77 (0.68)	
	Private enterprise, mean (SD)	3.61 (0.66)	2.74 (1.03)	3.63 (0.84)	2.83 (0.86)	3.43 (0.97)	3.70 (0.65)	
	Individuals, mean (SD)	3.65 (0.77)	3.06 (1.04)	3.61 (0.92)	3.07 (0.85)	3.52 (1.02)	3.58 (0.74)	
	Others, mean (SD)	3.25 (0.98)	2.89 (1.07)	3.33 (1.02)	2.97 (0.92)	2.98 (0.98)	3.21 (0.91)	
	*F* test (*df*)	3.508 (871)	1.570 (871)	6.512 (871)	2.946 (871)	8.666 (871)	7.882 (871)	
	*P* value	.009	.18	<.001	.02	<.001	<.001	
**Frequency of medical treatment**								
	0 times, mean (SD)	3.60 (0.85)	3.01 (1.17)	3.67 (0.94)	2.83 (0.87)	3.50 (1.05)	3.62 (0.79)	
	1-2 times, mean (SD)	3.67 (0.70)	2.87 (0.99)	3.69 (0.82)	2.92 (0.87)	3.65 (0.88)	3.66 (0.68)	
	3-4 times, mean (SD)	3.61 (0.72)	2.87 (1.10)	3.80 (0.84)	2.82 (0.85)	3.59 (0.94)	3.63 (0.70)	
	5 times and more, mean (SD)	3.79 (0.69)	3.02 (1.10)	3.94 (0.83)	2.63 (0.86)	3.64 (1.03)	3.85 (0.68)	
	*F* test (*df*)	3.216 (872)	1.406 (872)	5.119 (872)	5.636 (872)	0.865 (872)	5.606 (872)	
	*P* value	.02	.24	.002	.001	.46	.001	
**Health insurance**								
	Urban employee medical insurance, mean (SD)	3.74 (0.68)	3.04 (1.06)	3.89 (0.80)	2.76 (0.86)	3.74 (0.95)	3.78 (0.66)	
	Urban residents’ medical insurance, mean (SD)	3.64 (0.69)	2.80 (1.05)	3.64 (0.83)	2.80 (0.85)	3.49 (0.91)	3.67 (0.69)	
	New rural cooperative medical care, mean (SD)	3.34 (1.09)	2.59 (1.13)	3.63 (1.15)	2.89 (1.15)	3.10 (1.24)	3.28 (0.97)	
	Commercial health insurance, mean (SD)	3.00 (1.14)	3.03 (1.13)	3.07 (1.40)	3.75 (0.87)	3.10 (1.35)	3.26 (1.16)	
	None, mean (SD)	3.89 (1.04)	3.41 (1.23)	4.09 (0.87)	2.80 (0.82)	3.86 (1.08)	3.74 (0.90)	
	Others, mean (SD)	3.67 (0.47)	3.83 (0.24)	3.50 (0.71)	2.88 (0.53)	3.50 (0.71)	3.94 (0.09	
	*F* test (*df*)	2.042 (870)	4.705 (870)	4.350 (870)	2.637 (870)	3.708 (870)	4.182 (870)	
	*P* value	.16	<.001	.02	.02	.04	.02	

^a^2-tailed.

^b^1 CNY=US $0.14.

### Factors Associated With Satisfaction of Digital Medical Care

Hierarchical multiple regression analysis was conducted to evaluate the impact of each dimension of perception factors on older adults’ satisfaction with digital health care (as shown in Table 7). This analysis also assessed whether the effects of control variables on the explanatory variables were significant. The findings from this regression analysis provided valuable insights for constructing the subsequent structural equation model. Using demographic variables such as gender, age, and education as control variables, the model’s significance level was less than .001, and each variance inflation factor was below 5, indicating no multicollinearity among the variables and that the findings were statistically significant. All dimensions significantly influenced satisfaction with digital health care access, supporting hypothesis H5, which states that “behavioral intention positively influences satisfaction.” Demographic variables did not have a significant effect and were therefore excluded from the structural equation model construction. Regression analysis was conducted to confirm the reliability of the results after removing the control variables (model 3). The findings showed that the significance and direction of the coefficients for the core independent variables remained consistent, indicating that the model passed the robustness test and confirming a generally reliable relationship between these variables and the dependent variable (Table 7).

**Table 7:** Hierarchical multiple regression results.

Variables	Satisfaction		
	Model 1	Model 2	Model 3
Constant	3.931^a^	1.808^a^	1.570^a^
Gender	0.012	–0.041	—^b^
Age	–0.181^a^	0.008	—
Education	0.032	–0.042	—
Marriage	0.036	0.033	—
Living conditions	–0.110^a^	–0.035	—
Monthly disposable income	0.080^c^	–0.003	—
Previous occupations	–0.094^a^	–0.029	—
Frequency of medical treatment	0.055^c^	0.021	—
Health insurance	–0.021	–0.027	—
External variables	—	0.346^a^	0.354^a^
Perceived usefulness	—	0.110^a^	0.124^a^
Perceived ease of use	—	0.136^a^	0.114^a^
Perceived risk	—	–0.069^a^	–0.076^a^
Behavioral intention	—	0.069^c^	0.066^c^
*F* test (*df*)	10.893^a^ (866)	130.349^a^ (861)	159.84^a^ (870)
*R* ^2^	0.102	0.489	0.479
*ΔR* ^2^	0.102	0.387	—

^a^*P*<.001.

^b^Not applicable.

^c^*P*<.05.

### Structural Equation Modeling of Perceptions and Satisfaction of Digital Medical Care for Older Adults

**Figure 4:**
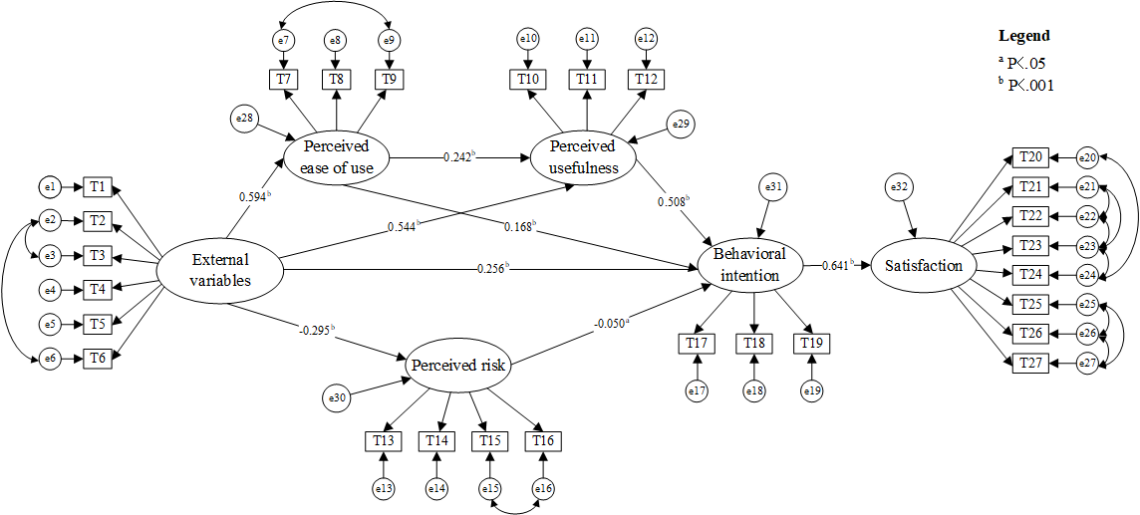
The structural equation model.

To determine the path coefficient relationships between the latent variables and the multiple mediation effects in this study, structural equation modeling was conducted using Amos 26.0 software (Figure 4). The significance of the paths was analyzed using *t* test results (Table 8). The test results are as follows:

- The significant effects of external variables on the dimensions, in descending order, were perceived ease of use (β=.594, *P*<.001), perceived usefulness (β=.544, *P*<.001), perceived risk (β=–.295, *P*<.001), and behavioral intention (β=.256, *P*<.001). This supports hypothesis H1.
- Perceived usefulness (β=.508, *P*<.001), perceived ease of use (β=.168, *P*<.001), and external variables (β=.256, *P*<.001) have significant positive effects on behavioral intention, while perceived risk (β=–.05, *P*=.04) has a weak negative effect. The effect of perceived usefulness is notably greater than that of the other dimensions. Therefore, hypothesis H2 is supported.
- Behavioral intention significantly and positively affects the satisfaction with digital health care access among older adults, with a path coefficient of 0.641 (*P*<.001). Therefore, hypothesis H5 is supported.

**Table 8:** Scalar estimates of the research model.

Outcome variables and predictive variables		*R* ^2^	β	SEs	Critical ratio	*P* value
**Perceived ease of use**						
	External variables	0.353	.594	0.042	18.801	<.001
**Perceived usefulness**						
	External variables	0.511	.544	0.038	14.352	<.001
	Perceived ease of use	—^a^	.242	0.028	6.714	<.001
**Perceived risk**						
	External variables	0.087	–.295	0.042	–7.945	<.001
**Behavioral intention**						
	External variables	0.703	.256	0.042	7.179	<.001
	Perceived ease of use	—	.168	0.025	5.87	<.001
	Perceived usefulness	—	.508	0.041	14.281	<.001
	Perceived risk	—	–.05	0.025	–2.087	.04
**Satisfaction**						
	Behavioral intention	0.41	.641	0.023	20.703	<.001

^a^Not available.

### Mediating Role Played by Perceived Ease of Use, Perceived Usefulness, and Perceived Risk

To verify the multiple mediating roles of perceived ease of use, perceived usefulness, and perceived risk between external variables and behavioral intention (H3) and the chained mediating roles of perceived ease of use and perceived usefulness (H4), the bias-corrected percentile bootstrap procedure test for the significance of each path of the multimediation model was performed using Amos 26.0, with 5000 repeated extractions to obtain 95% CI [46]. As can be seen from Table 9, the bootstrap (95% CI) of ind3 is [0-0.033], which contains 0, indicating that the mediation effect of this path is not significant, and the bootstrap (95% CI) of ind1, ind2, and ind4 does not contain 0, which has a significant mediation effect, and the value of the mediation effect is ind2>ind1>ind4. Thus, hypothesis H3 partially holds, the mediating effects of perceived ease of use and perceived usefulness are significant, the mediation hypothesis of perceived risk does not hold, and hypothesis H4 of chained mediation effects is valid.

**Table 9:** Results of distal mediation test.

Effect types	Effect	Boot SE	Boot lower level of confidence interval	Boot upper level of confidence interval	Ratio of indirect to total effect, %	Ratio of indirect to direct effect, %
Total effect	0.721	0.027	0.662	0.771	—^a^	—
Direct effect	0.256	0.045	0.169	0.345	35.5	—
Total indirect effect	0.465	0.033	0.403	0.537	64.5	—
ind1: external variables → perceived ease of use → behavioral intention	0.1	0.019	0.066	0.142	13.9	39.1
ind2: external variables → perceived usefulness → behavioral intention	0.277	0.034	0.213	0.348	38.4	108.2
ind3: external variables → perceived risk → behavioral intention	0.015	0.008	0	0.033	2.1	5.9
ind4: external variables → perceived ease of use → perceived usefulness → behavioral intention	0.073	0.016	0.044	0.11	10.1	28.5
C1: ind1 – ind2	–0.176	0.044	–0.266	–0.092	—	—
C2: ind1 – ind3	0.086	0.021	0.047	0.129	—	—
C3: ind1 – ind4	0.027	0.028	–0.027	0.081	—	—
C4: ind2 – ind3	0.262	0.036	0.197	0.336	—	—
C5: ind2 – ind4	0.203	0.041	0.124	0.286	—	—
C6: ind3 – ind4	–0.058	0.019	–0.098	–0.023	—	—

^a^Not available.

## Discussion

### Principal Findings

This study used a mixed methods approach, combining qualitative and quantitative analyses. Both methods revealed that while older adults recognize the convenience of digital access, their willingness to use digital devices is hindered by several factors. These include low literacy, vision and hearing impairments, inadequate assistance and guidance, and apprehension about new technologies. Additionally, there is a desire for digital devices to be adapted to the needs of older adults to enhance their satisfaction. The qualitative research provided insights into the digital challenges faced by older adults and highlighted their expectations for future digital development based on their personal needs, offering a scientific basis for designing the questionnaire. Meanwhile, the quantitative study utilized the questionnaire data to verify whether these challenges affected older adults’ willingness to use digital technologies and to assess the pathways and extent of this impact, thereby validating the hypotheses.

The current situation and trend in digital medical care for older adults reveal low awareness, low utilization, and high demand [47]. This study, based on the AM, examined the current state of digital medical services for older adults and explored the factors influencing their behavioral intention and satisfaction. The following conclusions, consistent with previous studies, were drawn:

Each dimension significantly affects behavioral intention. Jiao [48] argued that perceived ease of use has the greatest impact on the attitude toward continued use of online appointment platforms, while Shang and Wang [49] found that perceived usefulness positively influences the behavioral intention to use digital health care services among older adults. In this study, perceived usefulness emerges as the most critical factor influencing behavioral intention toward digital health care platforms and devices among older adults. This suggests that if digital health care services are designed to effectively serve and facilitate older adults, many other challenges can be overcome. Therefore, attention should be focused on transforming and upgrading digital health care platforms and devices to enhance service convenience and effectiveness. To encourage older adults to use digital health care devices, it is essential to balance the improvement of external variables—such as social support and the user-friendliness of the technology—with enhancing perceived usefulness and reducing perceived risk. This involves institutionalizing support, redesigning services, and refining the digital health care experience to accommodate the aging population effectively.

Pavlou [36] argued that perceived risk negatively affects the willingness to use technology, and this study similarly validates that finding. However, older adults perceive digital access to health care as low risk and believe it has minimal impact on their behavioral intentions. This study suggests the following reasons for this phenomenon: First, older adults often lack awareness of the potential risks associated with digitalization in health care, leading to limited concern about these risks. Second, most digital health care services are provided by public hospitals, and older adults’ trust in government-run health care organizations extends to their online platforms and self-service devices. Third, digital access platforms and devices are subject to uniform and stringent regulatory standards, which reduces negative press and fosters greater trust among older adults. Fourth, the convenience offered by digitalization sufficiently outweighs its potential risks, leading older adults to remain willing to experiment with and adapt to digital health care services.

Numerous studies have confirmed the positive effect of satisfaction on behavioral intention [50,51], which aligns with the common understanding that older adults’ satisfaction with various aspects of digital health care access influences their behavioral intentions. However, this study also explored whether older adults’ internal acceptance of digital health care predominantly determines their satisfaction with these services. This study argues that fundamentally improving digital health care services for older adults must begin with enhancing their willingness to accept and use digital health care platforms and devices. This approach aims to realize the positive, cumulative effects of behavioral intention and satisfaction in a cyclic manner.

Based on the study’s results, we propose strategies to enhance the willingness and satisfaction of urban older adults with digital health care services. These recommendations offer valuable insights for optimizing digital medical services in Hangzhou and for the development of similar health care initiatives in other cities.

### Improve Accessibility of Digital Medical Information and Assistance

The study results indicate that external variables, such as social environment factors and convenience, are significantly positively correlated with the perceived usefulness and ease of use of digital medical services among older adults. Convenience and related factors notably influence older adults’ health care behavior choices [52]. Therefore, it is crucial for various departments to collaborate in expanding and enhancing channels that improve the convenience and accessibility of digital health care knowledge and guidance for older adults. Government departments should leverage both traditional and new media channels to disseminate information about digital health care, enhancing public awareness and trust in government-provided services [53]. This approach can help reduce older adults’ perceived risks associated with digital health care, improve their perception of its usefulness, and thereby boost behavioral intentions. Additionally, community organizations should support these efforts by promoting local digital access platforms, acting as an auxiliary force to further engage and assist older adults. Older adults who have used intelligent platform terminals for senior care services should be encouraged to share their experiences and feelings, promoting positive perceptions of these platforms. Communities should organize digital access training sessions to provide more opportunities for older adults to become familiar with and utilize digital platforms in their daily lives. Teaching older adults how to identify beneficial information and basic cybersecurity skills will boost their confidence in using digital medical devices and improve their perceived ease of use [54]. Health care institutions should recruit more trained volunteers to offer digital access support, addressing the reluctance of older adults to use self-service machines due to a lack of timely assistance and guidance. While promoting digital access, it is crucial to accommodate the specific needs of older adults by providing alternative options such as green channels for manual services or one-on-one assistance from volunteers. In addition, fostering “interaction with relatives” and “interaction with friends” is essential, as these individuals can play an irreplaceable role in helping older adults overcome the “health digital divide” [55]. Such measures aim to enhance older adults’ perceptions of digital health care through external support, thereby encouraging the use of digital services, sharing the benefits of digital health care, and bridging the “digital divide in health.”

### Promote Digital Access Platform More Suitable for Older Adults

The most direct aspects of digital medical treatment that users experience are the interactive interface, operation process, and server response speed of apps and self-service machines. The quality of both hardware and software significantly impacts users’ ability to effectively utilize digital technologies [56]. Older adults may abandon the use of digital health services if they do not receive timely assistance when issues arise, exacerbating the health digital divide. Departments should focus on distributing resources for digital medical services more equitably and adapt mobile platforms and self-service machines to better meet the needs of older adults. To improve perceived ease of use and reduce perceived risk, the government should facilitate the unification of hospitals onto a single official platform for registration, payment, and other medical activities. At the same time, the credibility of an official platform will also boost older adults’ willingness to use digital medical services. Developers of smart terminals should optimize product design to cater to the characteristics of older adults, creating more user-friendly electronic products, apps, and websites. Programs should minimize unnecessary upgrades, be accessible to users with low education levels and older adults, enhance perceived ease of use, and ultimately improve behavioral intention and satisfaction.

### Increasing the Acceptance of Digital Health Services

Based on the findings from the questionnaire and interviews, the study identified that the barriers older adults face in digital medical processes are not limited to technical, social, and literacy issues, but also include personal and cognitive factors [1]. Addressing these barriers is essential for bridging the health digital divide for this population. As a result of financial, physical, and ideological factors, older adults often resist digital medical care [48]. Therefore, older adults should actively embrace technological advancements, shift their mindset away from fear and reluctance, and take the initiative to learn and master digital knowledge. By overcoming their hesitance and engaging with digital tools, they can foster a positive change in their ability to use these technologies effectively. Older adults should actively participate in digital training activities to enhance their skills in screening information, managing risks, and reducing perceived risk. This engagement will help improve their behavioral intentions and increase their satisfaction with digital medical care.

### Limitations

This study has several limitations. First, due to constraints related to time and resources, the survey focused solely on older adults aged 60 years and above in urban areas of Hangzhou. Future research should aim to increase the sample size and broaden the geographic scope to encompass a national population, thereby enhancing the representativeness of the findings. Second, while this study focused on 5 key factors—external variables, perceived usefulness, perceived ease of use, perceived risk, and behavioral intention—many other factors could influence willingness and satisfaction with digital medical services. Future studies could explore additional hypotheses and contributing factors to provide a more comprehensive understanding of these variables.

Ongoing advancements in internet technology and aging transformation are expected to shift older adults’ digital medical behaviors toward online health management, chronic disease management, online hospitals, and various other health services. This research lays a foundational basis for future studies on a broader range of digital health topics, aiming to help older adults bridge the digital health gap. For example, efforts can be directed toward assisting older adults with mobility impairment, disability, and incapacitation in accessing online medical consultations, purchasing medication, booking home appointments, and enhancing their ability to participate in and benefit from the digital society. Such initiatives are crucial for advancing the concept of active aging and ensuring that older adults globally can fully engage with and benefit from digital advancements.

### Conclusions

In summary, this study constructed a structural equation model to elucidate the effects and mechanisms of external variables, perceived usefulness, perceived ease of use, and perceived risk on behavioral intention and satisfaction with digital health care among older adults. To enhance the behavioral intention to use digital health care and increase satisfaction, it is crucial to optimize external influencing factors, improve perceived usefulness and ease of use, and reduce perceived risk. Achieving these goals requires concerted efforts from the government, various sectors of society, and the older adults themselves. By doing so, digital health care services can be better tailored to the needs of older adults, thereby improving their satisfaction, ensuring equitable access to health care services, and bridging the digital health divide.

## Appendix

Multimedia Appendix 1 Study questionnaire.

## Abbreviations

mHealth: mobile health
TAM: Technology Acceptance Model

## Data Availability

All data generated or analyzed during this study are included in this published article and its supplementary information files.
