# Supplementary material for: Perceptions and Satisfaction With the Use of Digital Medical Services in Urban Older Adults of China: Mixed Methods Study
Source: J Med Internet Res. 2024 Sep 20;26:e48654. doi: 10.2196/48654 (PMC11452758; doi:10.2196/48654)
Supplement: Multimedia Appendix 1 [file jmir_v26i1e48654_app1.docx]

**Questionnaire on digital medical behavior and satisfaction**

**among urban older adults**

Hello! Thank you very much for participating in this questionnaire!

We are students of health service management in Hangzhou Normal University, this is a questionnaire on the study of strategies to improve the digital friendliness of medical services for the urban older adults. This questionnaire is anonymous and the information you fill in will only be used for this study and will not be used for any commercial purposes, so please answer in peace.

Thanks again for your support and cooperation!

Note: In this questionnaire, "digital medical equipment" includes all mobile applications and hospital self-service machines that can provide online registration, billing, and inquiry services.

**Basic personal information**

1. Gender：□male □female
2. Age： years
3. Education： □Primary and below □Junior high school □Technical secondary school/ Senior high school □Junior college/ Bachelor and above
4. Marriage：□Married □Unmarried □Bereaved spouse
5. Living situation：□Living only with spouse □Living with spouse and children

□Living alone □Others

1. Your monthly disposable income (CNY)：

□<1000 □1001-3000 □3001-5000 □5001-7000 □>7000

1. Previous Occupations:

□Government Offices □Enterprises and Businesses □Private enterprise □Individuals □Others

1. Frequency of medical treatment in the past six months：

□0 times □1-2 times □3-4 times □5 times and more

1. What kind of health insurance do you have?

□Urban employee medical insurance □Urban residents' medical insurance □New Rural Cooperative Medical Care □Commercial Health Insurance □None □Others

1. Deterioration of physical function (e.g., vision loss, hearing loss) that affects your ability to use digital medical devices

□Totally disagree □Comparatively disagree □Generally

□Comparatively agree □Totally agree

1. Which registration methods do you prefer?

□Online registration □Self-service machine registration □Manual registration  □The receiving doctor makes an appointment for you at the next clinic.

□Make a call □Others（Please specify）

1. Which of the following problems do you see with digital medical equipment?

□Time limit is too short □Too complicated interface and process □Font size too small □Others（Please specify）

**Survey on the digital medical behavior of urban older adults** (Please answer the following questions according to your actual situation or real feelings, and tick the column that matches your view.)

| **The following questions are your attitude towards digital medical services.** | Totally disagree | Comparatively disagree | General | Comparatively agree | Totally agree |
| --- | --- | --- | --- | --- | --- |
| 1.I am willing to accept digital access to medicine. | 1 | 2 | 3 | 4 | 5 |
| 2.I would like to learn about the use of digital medical equipment. | 1 | 2 | 3 | 4 | 5 |
| 3.I would like to use digital medical equipment. | 1 | 2 | 3 | 4 | 5 |

| **The following questions are your thoughts on the effectiveness of digital medical services.** | Totally disagree | Comparatively disagree | General | Comparatively agree | Totally agree |
| --- | --- | --- | --- | --- | --- |
| 4.Digital access can save time. | 1 | 2 | 3 | 4 | 5 |
| 5.Digital access helps me get more information about medical care in advance. | 1 | 2 | 3 | 4 | 5 |
| 6.It is easier to adapt to society if you have digital medical skills. | 1 | 2 | 3 | 4 | 5 |
| **The following questions are your thoughts on the ease of digital medical services.** | Totally disagree | Comparatively disagree | General | Comparatively agree | Totally agree |
| 7.I can use my smartphone proficiently. | 1 | 2 | 3 | 4 | 5 |
| 8.I can make online appointments and payments. | 1 | 2 | 3 | 4 | 5 |
| 9.I can operate the self-service machines in the hospital. | 1 | 2 | 3 | 4 | 5 |

| **The following questions are your thoughts on the risks of digital health services.** | Totally disagree | Comparatively disagree | General | Comparatively agree | Totally agree |
| --- | --- | --- | --- | --- | --- |
| 10.I am concerned that third-party platforms used in the digital medical process (e.g., WeChat, various apps, etc.) may disclose personal information. | 1 | 2 | 3 | 4 | 5 |
| 11.I am concerned about the security of online and self-service machines payment. | 1 | 2 | 3 | 4 | 5 |
| 12.I am concerned that there is a haphazard fee for doctor's office billing. | 1 | 2 | 3 | 4 | 5 |
| 13.I am concerned about the accuracy of the self-service report retrieval. | 1 | 2 | 3 | 4 | 5 |
| **The following questions are perceptions of social and environmental factors influencing digital medical behavior.** | Totally disagree | Comparatively disagree | General | Comparatively agree | Totally agree |
| 14.If digital devices improve friendliness (e.g., longer operation time, simplified interaction interface and flow, enlarged fonts, etc.), it will increase my using willingness. | 1 | 2 | 3 | 4 | 5 |
| 15.The community offers training on the use of digital medical devices, and I would like to attend. | 1 | 2 | 3 | 4 | 5 |
| 16.I would like to read and study the digital medical information provided by the community. | 1 | 2 | 3 | 4 | 5 |
| **The following questions are your thoughts on how convenience affects digital medical behavior.** | Totally disagree | Comparatively disagree | General | Comparatively agree | Totally agree |
| 17.I think self-service registration (self-service machines or cell phone) is more convenient than manual registration. | 1 | 2 | 3 | 4 | 5 |
| 18.I think it is more convenient to make an appointment than to wait for a consultation on site. | 1 | 2 | 3 | 4 | 5 |
| 19.I think self-service payment (self-service machines or cell phone) is more convenient than tollbooth. | 1 | 2 | 3 | 4 | 5 |

**Satisfaction Survey of Digital Access Process** (Please answer the following questions according to your actual situation or true feelings, and put a tick in the column that matches your view.)

|  | Highly Dissatisfied | Dissatisfied | Common | Satisfied | Highly Satisfied |
| --- | --- | --- | --- | --- | --- |
| 1.Various ways of digital appointment registration | 1 | 2 | 3 | 4 | 5 |
| 2.Guidance on the use of digital medical equipment | 1 | 2 | 3 | 4 | 5 |
| 3.Online doctor appointment | 1 | 2 | 3 | 4 | 5 |
| 4.Call information indicator screen in the waiting room | 1 | 2 | 3 | 4 | 5 |
| 5.Pay for medical treatment in the doctor's office without paying at the tollbooth. | 1 | 2 | 3 | 4 | 5 |
| 6.Self-service payment (mobile phone/self-service machine) | 1 | 2 | 3 | 4 | 5 |
| 7.Self-service report retrieval (mobile phone/self-service machine) | 1 | 2 | 3 | 4 | 5 |
| 8. The whole process of digital medical treatment | 1 | 2 | 3 | 4 | 5 |

This is the end of the survey. Once again, I want to thank you for your helpful collaboration and send you and your family my best wishes！
